# Supplementary material for: Novel Thermally Reduced Graphene Oxide Microsupercapacitor Fabricated via Mask—Free AxiDraw Direct Writing
Source: Nanomaterials (Basel). 2021 Jul 25;11(8):1909. doi: 10.3390/nano11081909 (PMC8401507; doi:10.3390/nano11081909)
Supplement: Supplementary file 1 [file nanomaterials-11-01909-s001.zip › nanomaterials-1311123-supplementary.pdf]

Novel Thermally Reduced Graphene Oxide Microsupercapacitor Fabricated via Mask—Free AxiDraw Direct Writing

V. M. Maphiri [1], G. Rutavi [1], N. F. Sylla [1], S. A. Adewinbi [2], O. Fasakin [1] and N. Manyala [1,*]

[1] Department of Physics, Institute of Applied Materials, SARChI Chair in Carbon Technology and Materials, University of Pretoria, Pretoria 0028, South Africa; vusanimuswamaphiri@gmail.com (V.M.M.); rutavigift@yahoo.com (G.R.); ntoufasylla@gmail.com (N.F.S.); fasakinoladepo@gmail.com (O.F.)
[2] Department of Physics, Osun State University, Osogbo, Osun State 210001, Nigeria; adeksaheed007@gmail.com
* Correspondence: ncholu.manyala@up.ac.za; Tel.: +27-12-420-3549; Fax: +27-12-420-2516

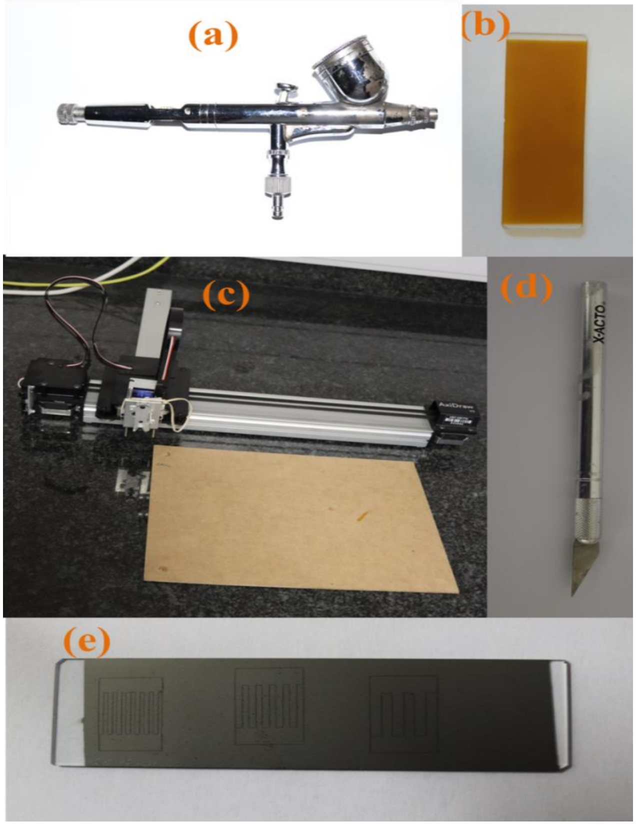

Figure S1. Optical image of (**a**) Spray gun, (**b**) GO thin film on microscopic glass (MSG), (**c**) axiDraw, (**d**) sharp blade pen and (**e**) TRGO patterned with μ-SC of various digits per unit area on microscopic glass.

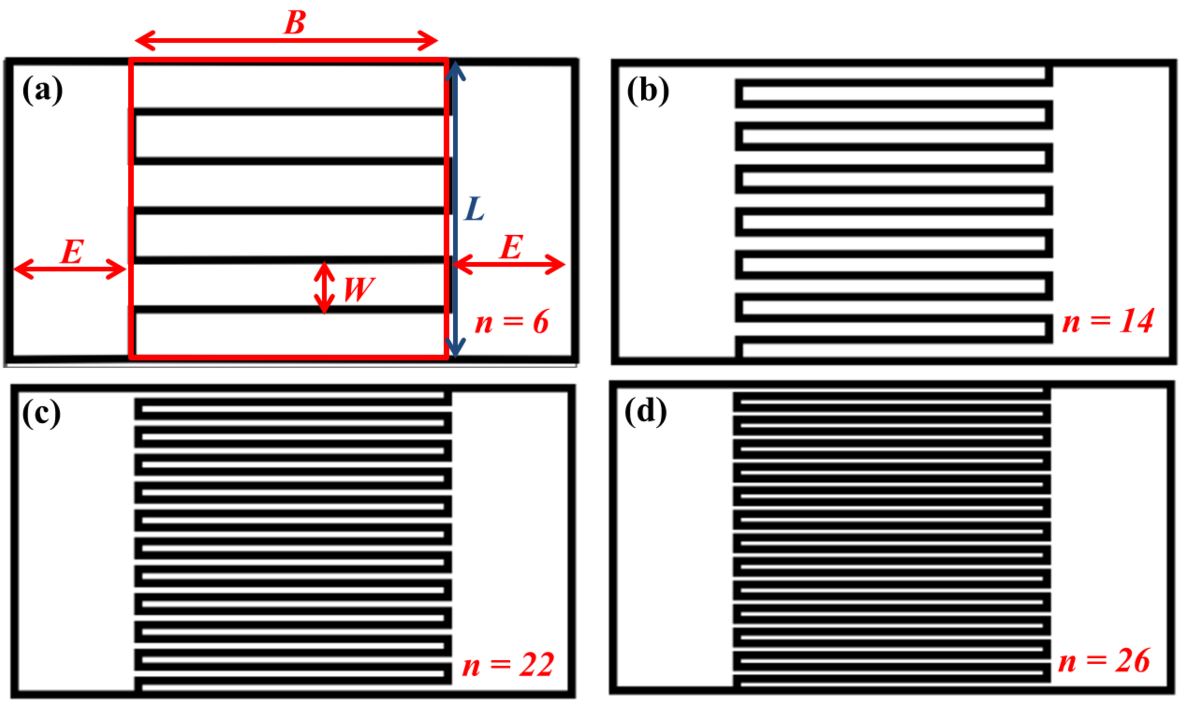

Figure S2. Inkscape blueprint schematic for the *n* (**a**) 6, (**b**) 14, (**c**) 22 and (**d**) 26 digits μ-SC.

The effective area was calculated using the formula (equation S1) derived below. The interspace area was subtracted from the total surface area. The interspace area has two components which is the horizontal (along the breadth) and vertical (along the length) component.

Effective area = Total surface area – Total interspace area

= Total surface area – (Horizontal interspace area + Vertical interspace area)

$= L \times B - ((n \times W \times i) + B \times (n - 1) \times i)$

$= L \times B - (i \times ((n \times W) + B \times (n - 1)))$

$$= L \times B - (i \times (L + B \times (n - 1)) \quad \text{(S1)}$$

Thus, Total interspace area is given by (equation S2)

$$i \times (L + B \times (n - 1)) \quad \text{(S2)}$$

Table S1. Dimensions and parameters of μ – SC (6), (14), (22) and (26).

**Parameters**	**μ-SC (6)**	**μ-SC (14)**	**μ-SC (22)**	**μ-SC (26)**
Number of digits per unit area, *n* (cm^{-2})	6	14	22	26
Width, *W* (mm)	1.66	0.71	0.45	0.38
Length, *L* and Breadth, *B* (mm)	10	10	10	10
Interspace, *i*(μm)	38	38	38	38
Edge, *E* (mm)	4	4	4	4
Total surface area (mm^2)*	100	100	100	100
Total interspace area(mm^2)	2.28	5.32	8.36	9.88
Effective area (mm^2)	97.72	94.68	91.64	90.12

*Total surface area and effective area doesn't include μ-SC edge area (see Fig. S7)

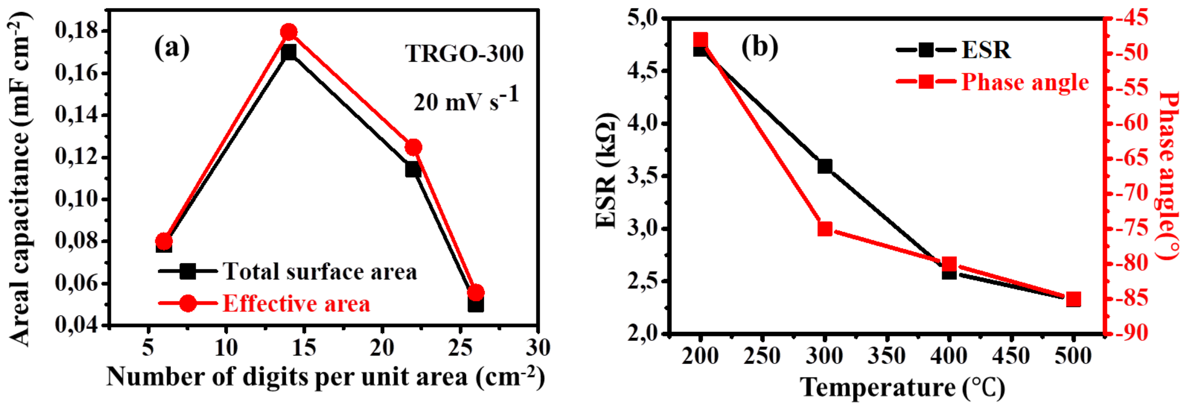

Figure S3. (**a**) Areal capacitance of the total surface area versus effective area at 6, 14, 22 and 26 digits per unit area (cm^{-2}) and (**b**) ESR and phase angle as a function of reducing temperature.

The CV curves of the μ-SC are displayed in Figs. S11 and S12 where (a)–(d) corresponds to the TRGO 200 to TRGO-500, respectively, measured at the potential window of 0–0.8V. The scan rate of these μ-SCs increased from 50 to 1000 mVs^{-1} as the reducing temperature increased from 200 to 500 °C. Thus, the scan rate is directly proportional to the reducing temperature.

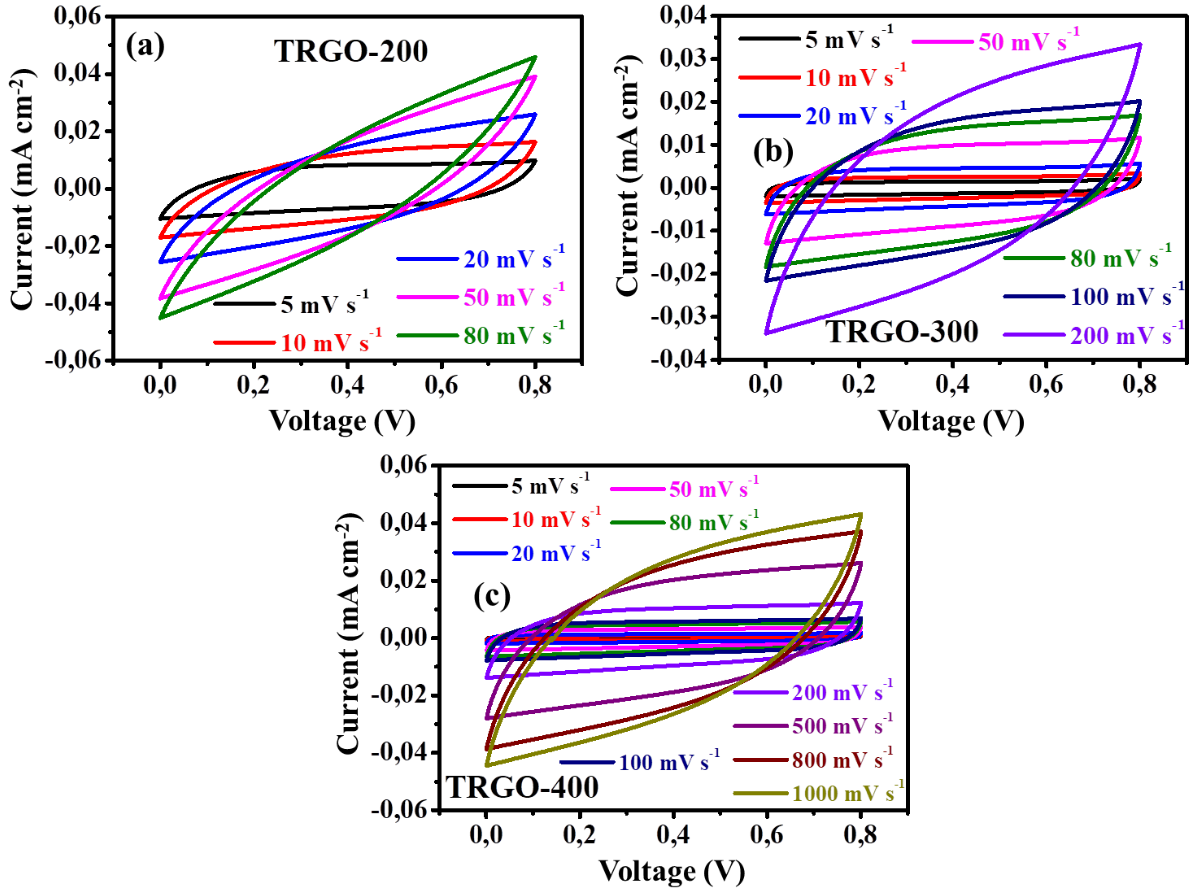

Figure S4. The CV curves at (**a**) TRGO-200, (**b**) TRGO-300, (**c**) TRGO-400 and (**d**) TRGO-500 at various scan rates.

Table S2. The areal capacitance determined from the CV curve.

Scheme 1.	Areal capacitance (mF cm^{-2})			
	**TRGO-200**	**TRGO-300**	**TRGO-400**	**TRGO-500**
5	0.7074	0.1451	0.0444	0.0319
10	0.5421	0.1227	0.0382	0.0298
20	0.3773	0.1061	0.0348	0.0274
50	0.2085	0.0865	0.0301	0.0256
80	0.1498	0.0756	0.0282	0.0244
100	-	0.0695	0.0267	0.0239
200	-	0.0506	0.0234	0.0220
500	-	-	0.0179	0.0194
800	-	-	0.0148	0.0172
1000	-	-	0.0140	0.0159

The area capacitance (C) was evaluated from the GCD curve by applying equation (S3) as shown below

$$C = \frac{I \times \Delta t}{\Gamma \times \Delta V} \quad \text{(S3)}$$

where, I is the discharge current, Δt is the discharge time, Γ is the area and ΔV is the voltage.

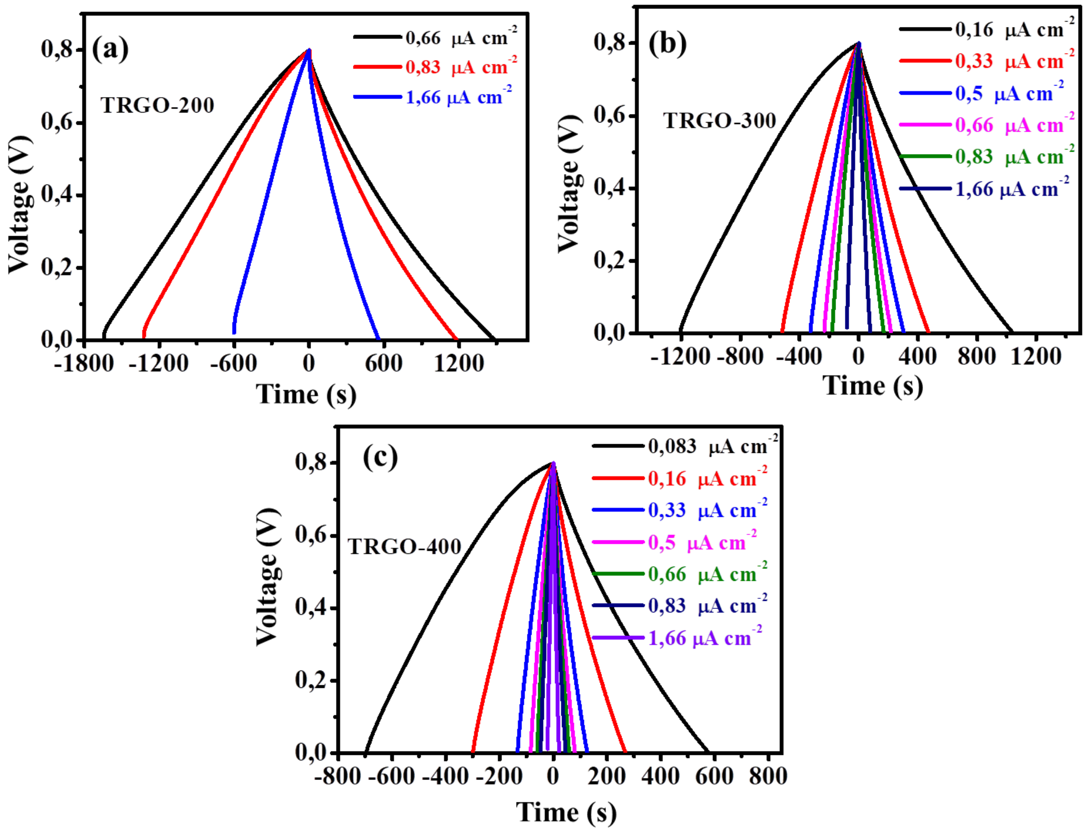

Figure S5. The GCD curves of μ-SC at different current densities for (a) TRGO-200, (b) TRGO-300, (c) TRGO-300 and (d) TRGO-500.

Table S3. The areal capacitance determined from the GCD curve.

Current density (μA cm^{-2})	Areal capacitance (mF cm^{-2})			
	**TRGO-200**	**TRGO-300**	**TRGO-400**	**TRGO-500**
0.083	-	-	0.0600	0.0387
0.16	-	0.2143	0.0536	0.0366
0.33	-	0.1952	0.0517	0.0367

0.5	-	0.1904	0.0501	0.0364
0.66	1.2255	0.1809	0.0481	0.0356
0.83	1.2262	0.1754	0.0472	0.0354
1.66	1.1569	0.1506	0.0434	0.0338

The µ-SCs of TRGO-200 to TRGO-500 delivered an areal power density ranging from 0.3316 to 0.3709 mW cm^{-2} corresponding to the areal energy density in the range of 0.1368 – 0.0017 mW h cm^{-2}. It is very clear that the delivered energy decrease as the reducing temperature increase while the power is increasing.

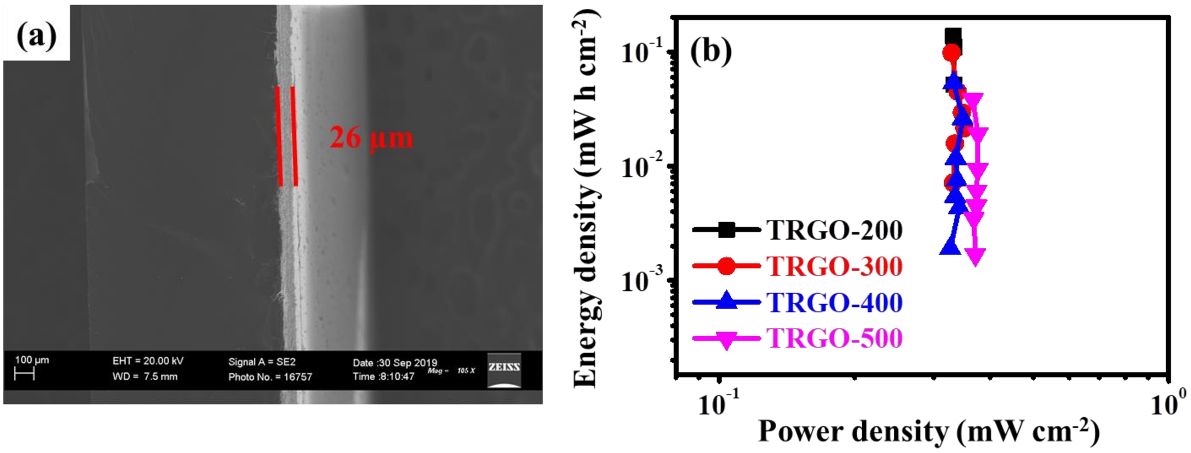

Figure S6. (**a**) SEM cross-sectional image of the TRGO-500 µ-SC and (**b**) Ragone plot of the TRGO-200, TRGO-300, TRGO-400 and TRGO-500.

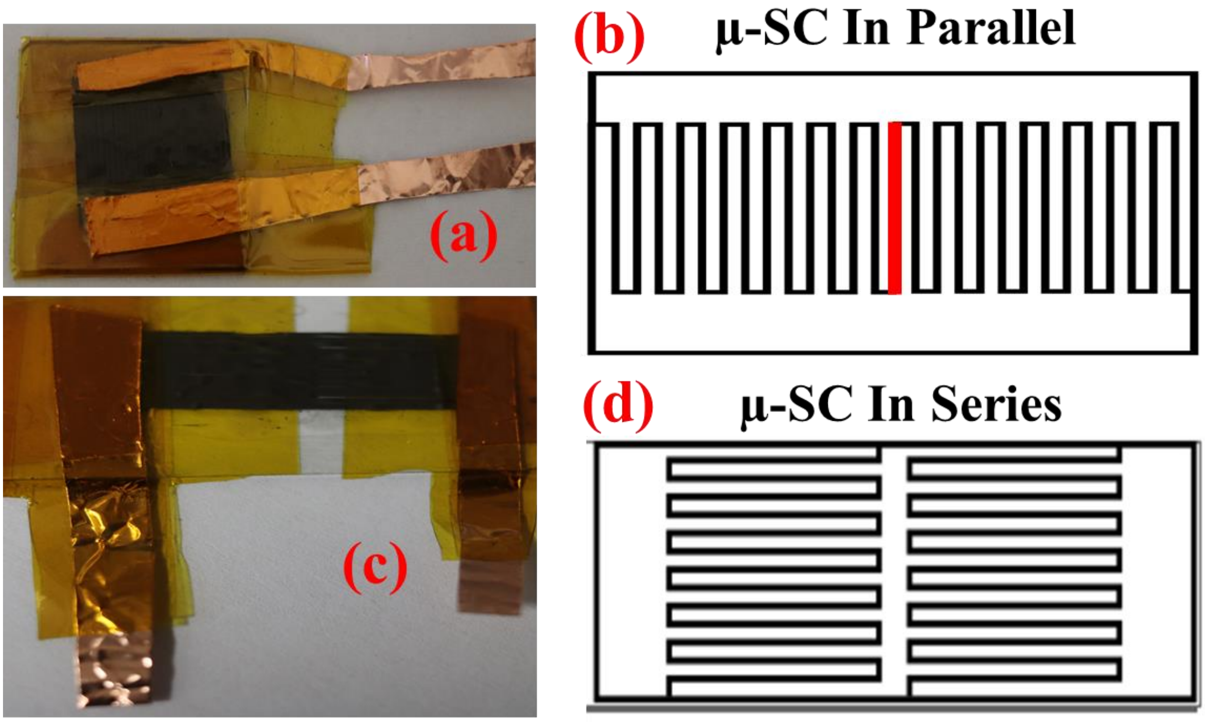

Figure S7. Digital photography of µ-SC in (**a**) parallel and (**c**) series. Inkscape blueprint of µ-SC in (**b**) parallel and (**d**) series.
